# Changes in phosphorus mobilization and community assembly of bacterial and fungal communities in rice rhizosphere under phosphate deficiency

**DOI:** 10.3389/fmicb.2022.953340

**Published:** 2022-08-03

**Authors:** Ruibo Sun, Wenjie Zhang, Yangbing Liu, Wenjing Yun, Bingbing Luo, Rushan Chai, Chaochun Zhang, Xingjia Xiang, Xiaofeng Su

**Affiliations:** ^1^Anhui Province Key Lab of Farmland Ecological Conservation and Pollution Prevention, Engineering and Technology Research Center of Intelligent Manufacture and Efficient Utilization of Green Phosphorus Fertilizer of Anhui Province, College of Resources and Environment, Anhui Agricultural University, Hefei, China; ^2^Key Laboratory of JiangHuai Arable Land Resources Protection and Eco-restoration, Ministry of Natural Resources, College of Resources and Environment, Anhui Agricultural University, Hefei, China; ^3^Anhui Provincial Territorial Space Planning Institute, Hefei, China; ^4^Anhui Province Key Laboratory of Wetland Ecosystem Protection and Restoration, School of Resources and Environmental Engineering, Anhui University, Hefei, China; ^5^Biotechnology Research Institute, Chinese Academy of Agricultural Sciences, Beijing, China

**Keywords:** phosphorus deficiency, rhizosphere microbes, microbial community assemblage, rice, P-mobilizing microbes

## Abstract

Rhizosphere microorganisms are closely associated with phosphorus (P) uptake in plants and are considered potential agents to mitigate P shortage. However, the mechanisms of rhizospheric microbial community assembly under P deficiency have yet to be elucidated. In this study, bacterial and fungal communities in rice rhizosphere and their P mobilization potential under high (+P) and low (−P) concentrations of P were investigated. Bacterial and fungal community structures were significantly different between −P and +P treatments. And both bacterial and fungal P-mobilizing taxa were enriched in-P treatment; however, the proportion of P-mobilizing agents in the fungal community was markedly greater than that in the bacterial community. A culture experiment confirmed that microbial phosphate solubilizing capacity was significantly higher in −P treatment compared with that in +P treatment. −P treatment lowered bacterial diversity in rice rhizosphere but increased fungal diversity. Further analysis demonstrated that the contribution of deterministic processes in governing bacterial community assembly was strengthened under P deficiency but was largely weakened in shaping the fungal community. These results highlighted that enriching P-mobilizing microbes in the rhizosphere is a vital way for rice to cope with P deficiency, and that fungi contribute considerably to P mobilization in rice rhizosphere. Findings from the study provide novel insights into the assembly of the rhizosphere microbiome under P deficiency and this will facilitate the development of rhizosphere microbial regulation strategies to increase nutrient uptake in plants.

## Introduction

Phosphorus (P) is crucial for plant growth as it is an indispensable element in DNA, RNA, ATP, phospholipids, and other compounds involved in metabolism. In agricultural systems, P in most soils is not sufficient to support the demand of high yield, thus, large amounts of P fertilizers are applied into the soil. Global consumption of total P fertilizer increased from 4.6 to 17.5 Tg P yr.^−1^ between 1961 and 2013 (Lu and Tian, 2017). However, P can react strongly with soil minerals, such as iron, aluminum, and calcium, forming complexes with low solubility, which lock up P and limit the bioavailability for plant uptake (Shen et al., 2011; Johnston et al., 2014). With the low use efficiency of P and increasing demands of food production, the requirement for P is predicted to increase by 50–100% by 2050 (Cordell et al., 2009). As a non-renewable resource, P will be a limiting factor in agricultural production in the near future (Cordell et al., 2009; Alewell et al., 2020). Thus, increasing P use efficiency is a potential way to relieve the problem of P shortage.

Soil microbes are strong regulators for P bioavailability, with some recognized as P mobilizers. Numerous soil microbes can mobilize insoluble P-complexes into a soluble form by solubilizing insoluble inorganic P (Pi) compounds and mineralizing organic P (Po; Adhya et al., 2015; Amadou et al., 2021). These P-mobilizing microbes are widely distributed in soil and exhibit high diversity (Li et al., 2021). Lowering soil pH and chelation are the two primary approaches used by microbes to solubilize Pi compounds. Pi-solubilizing microbes release organic acids (including dicarboxylic acids, aromatic acids, aliphatic acids, citric acids, and lactic acids) from metabolic activity, such as oxidative respiration or fermentation. These organic acids decrease soil pH and consequently enhance the dilution of Ca phosphates in alkaline soil (Devau et al., 2009). Organic acids can also act as “chelates,” reacting with Al^3+^ and Fe^3+^ to decrease the precipitation of Al and Fe phosphates (Kalayu, 2019). Mineralization of Po by phosphatases is another way that microbes increase P availability. Po comprises a substantial part (30–65%) of total P in soils, hence Po transformation greatly impacts P bioavailability (Amadou et al., 2021). With high potential in improving soil P availability, P-mobilizing microbes are considered an ecofriendly and economically sound approach to overcome P scarcity (Kalayu, 2019), and these microbes have been applied in the laboratory and experimental field (Shahid et al., 2015; Zhang et al., 2018; Chen and Liu, 2019; Liang et al., 2020). However, due to limited adaption and proliferation of these microbes in soil, it is difficult to achieve high levels of activity with the microbes in practical production.

Rhizosphere microbes are closely associated with nutrient uptake in plants. Zhang et al. (2019) revealed that a rice variety with high nitrogen use efficiency (NUE) recruited a root microbiota containing more genera with nitrogen metabolism functions compared with a variety with low NUE. This variation in root microbiota was associated with *NRT1.1B* (which encodes a nitrate transporter and sensor), indicating that regulation of rhizosphere microbiota composition and function is an auxiliary approach utilized by plants to increase nutrient uptake. Various P-mobilizing microbes in rhizosphere soil can improve plant P uptake and growth (Zhang et al., 2018; Wang et al., 2020; Tian et al., 2021), and the P-mobilizing microbes in the rhizosphere varied with plant growth stage (Li et al., 2019). Simultaneously, the rhizospheric microbiome also varied under different P levels. For example, Long and Yao (2020) found that fungi diversity in rice rhizosphere (*Oryza sativa* L.) was higher under high P input conditions, while Tian et al. (2020) reported that P fertilization suppressed some bacterial taxa, such as *Bacillales* and *Pseudomonadales*. In addition, Silva et al. (2017) found that microbial community composition in maize rhizosphere was associated with the type of P fertilizer used as these fertilizers have different solubilities. Furthermore, these studies conjectured a tendency that microbial taxa contributing to P mobilization were suppressed under high levels of P, but this hypothesis has not been reliably verified. Thus, elucidating the response of microbial functions associated with P mobilization to P deficiency is vital to understand the contribution of microbes in the ability of plants to cope with P deficiency.

Rhizosphere microbial assemblage are greatly shaped by plant roots, primarily through changing the soil environment and substrates by secreting root exudates (Bakker et al., 2013; Hu et al., 2018; Zhalnina et al., 2018). Root exudate composition varies markedly under different P concentrations. For example, low phosphate levels can increase exudation of ferulic acid, p-coumaric acid, and nicotinamide (Brisson et al., 2022). The changes in root exudate composition under P deficiency increase P availability through their Pi-solubilizing activity and altered root system architecture (Pantigoso et al., 2020), while also possibly changing rhizosphere microbial communities. *Arabidopsis* can modulate its root microbiota using specialized root triterpenes, which act as antibiotics or proliferating agents for root microbiota (Huang et al., 2019). This modulation suggests that manipulating rhizosphere microbiota is a supplementary mechanism that allows plants to cope with resource deficiency. Plant rhizosphere microbiota also vary considerably under different P concentrations (Long and Yao, 2020; Amadou et al., 2021). However, the taxonomic changes and differences in P mobilization of rhizospheric microbial taxa, including bacteria and fungi, in response to P deficiency have not been elucidated.

Although induration of Pi-solubilizing microbes has been found to be beneficial for increasing soil P availability and plant P uptake (Shahid et al., 2015; Zhang et al., 2018; Tian et al., 2021), and Pi-solubilizing microbes are a promising biofertilizer to mitigate P shortage (Kalayu, 2019), such findings were acquired in specialized experiments, and many challenges remain for widespread practical application (Alori et al., 2017). These challenges arise from the limited colonization of the plant rhizosphere by effective Pi-solubilizing strains due to competition from indigenous species and the selection of plant roots. Thus, understanding the mechanisms of rhizospheric microbial community assembly is of vital importance for the practical utilization of functional biofertilizer agents such as Pi-solubilizing microbes.

Many P-mobilizing microbes have been identified and isolated from the plant rhizosphere, and the roles of these microbes in P mobilization and growth enhancement in plants have been confirmed. However, the response and assembly mechanisms of the rhizosphere microbial community under P deficiency remain poorly characterized. This study explored the taxonomic composition, P-mobilization capacity, and assembly of bacterial and fungal communities in rice rhizosphere under different P levels. The work aimed to (1) compare the response of community composition and P-mobilization potential of bacteria and fungi under P deficiency; (2) investigate the potential assembly mechanisms of the microbial community involved in P solubilization in rice rhizosphere.

## Materials and methods

### Experiment design

A pot culture experiment comprising two different P contents was set up: −P, soil without P fertilization; +P, soil applied with P fertilizer. The amounts of nitrogen (N) and potassium (K) were kept constant in the two treatments. The contents of N and K were 115 mk/kg and 75 mg/kg, respectively, while the P content in −P and +P treatments was 0 and 60 mg/kg, respectively. N, P, and K fertilizers were applied as urea, KH_2_PO_4_, and KCl, respectively. Each treatment had three replicates (pots), and each replicate contained 600 g soil (dry weight).

After germination, six rice (*O. sativa* L. spp. Japonica, cv. Nipponbare) seedlings were transplanted into each pot and incubated in a phytotron with 14-h light at 30°C and 10-h dark at 24°C. The light intensity was 400 μmol m^−2^ s^−1^ and the relative humidity was 65–70%. Sterile deionized water was added to each pot once every 2 days to maintain a flooded condition.

Soil used in this study was Ferralic Cambisol, sampled from the Yingtan Red Soil Ecological Experimental Station of the Chinese Academy of Sciences in Jiangxi Province, China (28°12′ N, 116°55′ E). The soil was planted with rice for a long time without fertilization. Original properties of the soil were: available P (AP) 1.95 mg/kg, total P (TP) 0.50 mg/kg, pH 5.45, and soil organic matter (SOM) 1.36%.

### Rhizosphere soil and plant sampling and chemical analysis

Soil and plants were sampled after incubation for 30 days. The whole plant was carefully removed from the pot. The soil lightly adhered to the roots was carefully removed then the root was thoroughly washed with sterile double-distilled water. The spent regenerant was centrifuged and the supernatant was removed. The sediment was collected as rhizosphere soil. The washed root was collected for properties measurement.

The pH of the supernatant mentioned above was measured using a pH meter (Mettler Toledo FE28, Switzerland), then it was converted to the value subjected to a soil: water ratio of 1: 2.5 (weight/volume) based on soil weight, moisture content, and water volume. The transformed pH value was perceived as rhizosphere soils pH. Soil AP was extracted with 0.5 M NaHCO_3_ solution and then measured using the molybdenum blue method (Crouch and Malmstadt, 1967). For determination of TP content, soil was digested with NaOH then measured with the molybdenum blue method. SOM was measured using the dichromate oxidation method (Shaw, 1959).

After measuring fresh weight, the roots and aboveground parts of the rice plants were dried at 105°C. TP contents in stem and leaf were measured using the molybdenum blue method after digestion with H_2_SO_4_-H_2_O_2_.

### Determination of microbial Pi solubilizing ability

Pi-solubilizing abilities of rhizosphere microbes under different concentrations of P were determined by measuring the solubilization of Ca_3_(PO_4_)_2_. Rhizosphere microbes were extracted from 1 g rhizosphere soil mixed with 9 ml sterilized water. The soil/water mixture was centrifuged at 3000 rpm for 2 min, then 1 ml supernatant was added to 50 ml sterile PVK liquid medium [5 g Ca_3_(PO_4_)_2_, 0.5 g (NH_4_)_2_SO_4_, 0.1 g MgSO_4_, 0.2 g KCl, 10 g dextrose, 0.5 g yeast extract, 0.0001 g MnSO_4_, 0.0001 g FeSO_4_, and 1 l distilled water] and incubated for 5 days at 28°C and 180 rpm in a thermostatic shaker. A blank control with 1 ml sterilized water was included. Five millimeters of culture solution was removed at 0,12, 24, 48, 72, 96, and 120 h, and centrifuged at 5000 *g* for 2 min. P content in the supernatant was measured using inductively coupled plasma emission spectroscopy (ICP-OES, PerkinElmer Avio 200).

After incubation, microbes in each sample were collected for DNA extraction and high-throughput sequencing to identify Pi- solubilizing agents.

### DNA extraction, PCR, and high-throughput sequencing

Soil total DNA was extracted from 0.5 g soil using a FastDNA Spin Kit for Soil (MP Biomedicals, Santa Ana, CA, United States).

Bacterial and fungal community composition were investigated using high-throughput sequencing. Primer sets 799F/1193R (5′-AACMGGATTAGATACCCKG-3′; 5′-ACGTCATCCCCACCTTCC-3′; Chelius and Triplett, 2001; Bodenhausen et al., 2013) targeting the V5-V7 region of 16S rRNA gene, and ITS1f/ITS2 (5′-CTTGGTCATTTAGAGGAAGTAA-3′; 5′-GCTGCGTTCTTCATCGATGC-3′) targeting the ITS (internal transcribed spacer) of fungal rRNA (Walters et al., 2016) were used for the survey of bacterial and fungal communities, respectively. PCRs were performed in a 25-μl system containing 12.5 μl PCR premix (Ex Taq^TM^; Takara, Shiga, Japan), 0.5 μl (10 μm) of the forward and reverse primers (final concentration of each primer 0.2 pmol μl^−1^), 0.5 μl DNA template (20 ng), and 11 μl sterile double-distilled water. Thermocycle conditions comprised an initial denaturation at 94°C for 10 min, 30 cycles of denaturation at 94°C for 30 s, annealing at 55°C/56°C (16S rRNA/ITS) for 45 s, and extension at 72°C for 1 min, followed by a final extension at 72°C for 10 min (Sun et al., 2020, 2022).

After checking the quality of the PCR products, high-throughput sequencing was performed using the Illumina HiSeq2000 platform (Illumina, San Diego, CA, United States). The sequencing data have been deposited in the European Nucleotide Archive (accession no. PRJEB52856).

### Bioinformatic analysis of the high-throughput sequencing data

Bioinformatic analysis was performed according to previous studies with some modifications (Shi et al., 2019; Sun et al., 2022). VSEARCH (version 2.21.1) was used for the bioinformatic analysis of high-throughput sequencing data (Rognes et al., 2016). Paired-end reads were merged and then quality filtering was performed to obtain high-quality reads (expected errors per base <0.001, without N, length > 150 bp). Next, chimeric reads were detected and removed using the improved version of the UCHIME algorithm (uchime3_denovo; Edgar et al., 2011). After removing primers, the high-quality reads were further denoised using the UNOISE algorithm (version 3) and generated ZOTUs (zero-radius operational taxonomic units). Taxonomic assignment of the ZOTUs was performed using RDP Classifier (Wang et al., 2007) based on the reference of SILVA rRNA database (version 138) and UNITE database (version 8.3). ZOTUs that could not be assigned as Bacteria or Fungi were removed, and subsampling was performed to generate the rarefication tables of bacterial and fungal ZOTUs. Finally, all the samples contained 80,000 and 60,000 reads for bacterial and fungal ZOTU tables, respectively, which were used for downstream analysis.

PICTUSt2 (phylogenetic investigation of communities by reconstruction of unobserved states; Douglas et al., 2020) was used to predict the functional profiles of the bacterial community.

### Statistical analysis

Statistical analysis and figure drawing were performed using R (version 4.0.2) following our previous study (Sun et al., 2021, 2022). The Kruskal–Wallis rank sum test (Corder and Foreman, 2009) was used for the significance test of difference of variables between −P and +P. Principal coordinate analysis (PCoA) was performed based on Bray–Curtis distance using “vegan” library to show the difference of microbial communities under different P treatments. Analysis of similarities (ANOSIM) was used to check whether there is a significant difference between microbial communities under −P and +P treatments. Abundance-based beta-null deviation measure was used to test the roles of deterministic and stochastic processes in soil microbial community assembly (Tucker et al., 2016). A heatmap was drawn using “pheatmap” library, while other figures were drawn using “ggplot2” library.

## Results

### Effects of P treatment on rhizosphere soil properties and plant P uptake

AP, pH, and SOM in rhizosphere soil were significantly higher in +P treatment compared with in −P treatment. However, TP was not significantly different between −P and +P treatments (Table 1).

**Table 1:** Variation of soil and rice properties in −P and +P treatments.

Treatment	Soil				Plant					
	AP (mg/kg)	TP (g/kg)	pH	SOM (%)	RFW (g)	RDW (g)	SFW (g)	SDW (g)	Stem P (mg/kg)	Leaf P (mg/kg)
−P	2.10 ± 0.24b	0.62 ± 0.08a	4.84 ± 0.01b	1.87 ± 0.05b	0.38 ± 0.08b	0.07 ± 0.02b	7.80 ± 1.00b	2.50 ± 0.50b	0.29 ± 0.16b	0.15 ± 0.05b
+P	3.96 ± 0.30a	0.66 ± 0.09a	4.87 ± 0.02a	1.95 ± 0.05a	0.77 ± 0.18a	0.16 ± 0.01a	10.78 ± 1.39a	3.71 ± 0.58a	0.68 ± 0.19a	0.29 ± 0.09a

AP: available P; TP: total P; SOM: soil organic matter; RFW: root fresh weight; RDW: root dry weight; SFW: shoot fresh weight; SDW: shoot dry weight. Values are the average ± standard deviation (*n* = 3). Different letters indicate significant difference between −P and +P (*p* < 0.05).

Rice growth and P uptake were largely impacted by P addition. Root fresh weight (RFW), root dry weight (RDW), shoot fresh weight (SFW), and shoot dry weight (SDW) of rice were significantly greater in +P treatment compared with in −P treatment. However, P deficiency had a greater impact on rice root compared with that on rice shoot. RFW and RDW in +P treatment were more than twice that in −P treatment, but SFW and SDW only increased by 38.21 and 48.40%, respectively, under +P treatment. +P treatment also increased stem and leaf P contents, which were 134.48 and 93.33% higher, respectively, in +P treatment compared with −P treatment (Table 1).

### Variation in rhizosphere microbial communities under different P treatments

**Figure 1:**
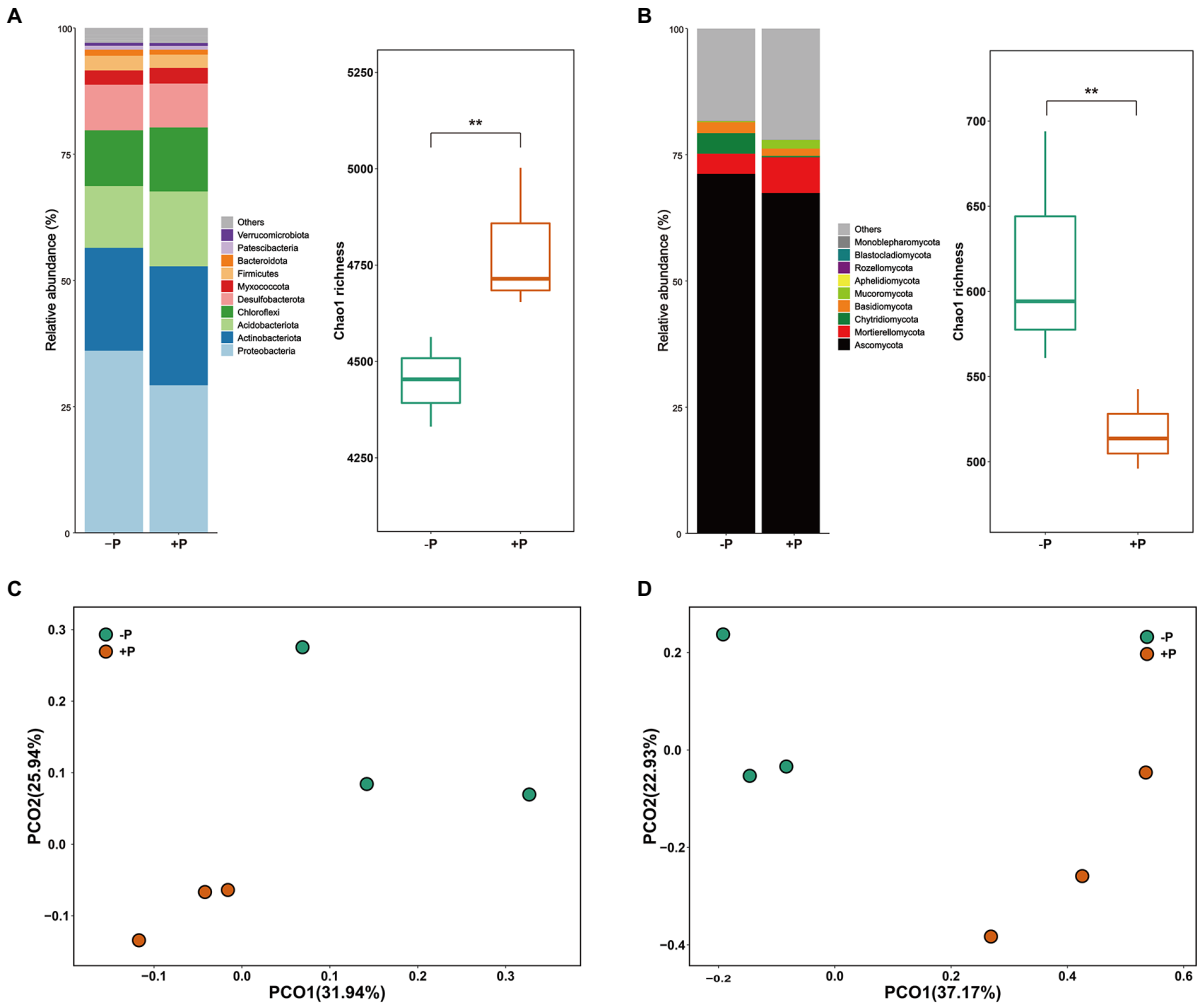
Community composition and diversity of bacteria **(A)** and fungi **(B)** under different concentrations of soil P. Asterisks indicate significant differences between −P and +P treatments (*p* < 0.01). PCoA plots showing the changes of soil bacterial **(C)** and fungal **(D)** communities under different concentrations of P.

Proteobacteria, Actinobacteriota, Acidobacteriota, Chloroflexi, Desulfobacterota, Myxococcota, Firmicutes, Bacteroidota, Patescibacteria, and Verrucomicrobiota were the dominant bacterial phyla in rice rhizosphere soil, accounting for 97.03% of the total reads (Figure 1A). Ascomycota, Chytridiomycota, Mortierellomycota, Basidiomycota, and Mucoromycota were the top five phyla (in relative abundance) of the fungal community, accounting for 79.78% of the total reads (Figure 1B). The bacterial and fungal communities varied between the two experimental treatments. Compared with +P treatment, −P treatment significantly enriched Proteobacteria but diluted Chloroflexi in the bacterial community, and significantly enriched Ascomycota and Chytridiomycota but diluted Mortierellomycota and Mucoromycota in the fungal community. The changes in bacterial and fungal communities were further depicted in the 2D PCoA plot, which showed a distinct separation of bacterial and fungal communities under the two different P treatments (Figures 1C,D). The results of ANOSIM showed that bacterial and fungal communities were significantly different between −P and +P treatments (Table 2).

**Table 2:** Results of ANOSIM of bacterial and fungal communities under −P and +P treatments.

Bacterial community		Fungi community	
R	*p*	R	*p*
0.548	0.008	0.601	0.007

Bacterial richness was significantly higher in −P treatment compared with that in +P treatment. However, the opposite result was observed for the fungal community with the −P treatment harboring higher diversity compared with the +P treatment (Figures 1A,B).

### Effects of P treatment on abundance of genes involved in P mobilization

**Figure 2:**
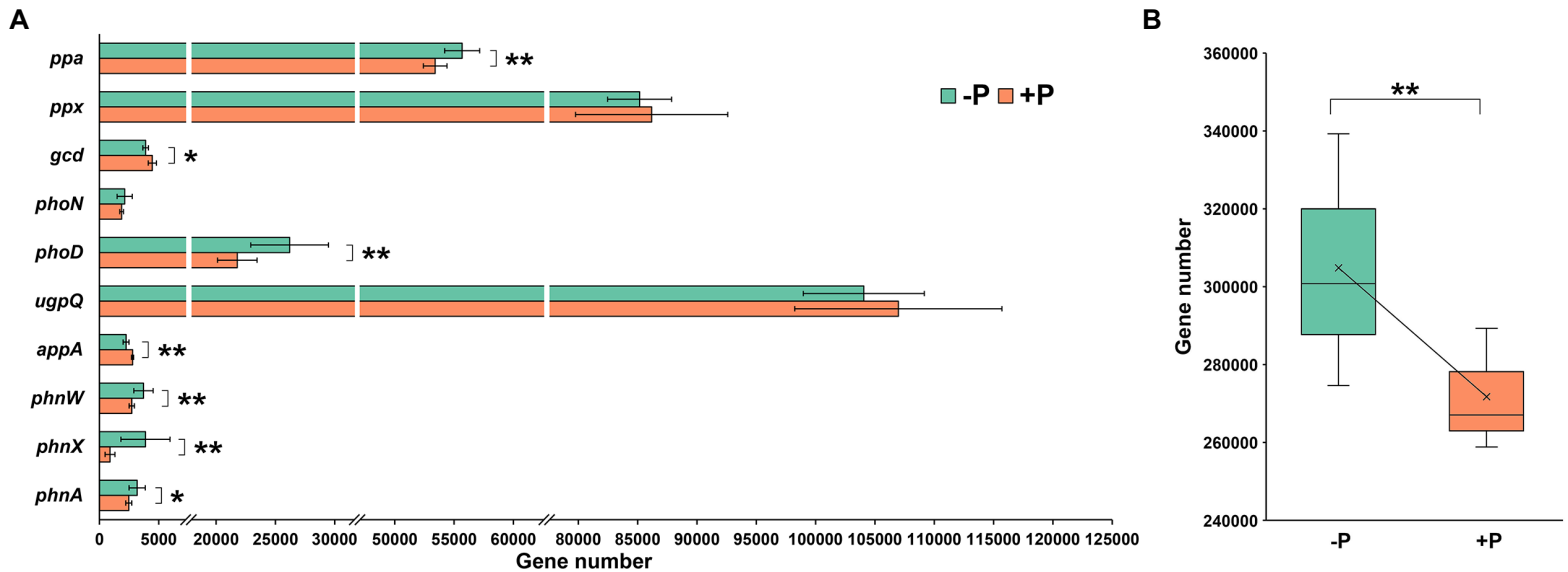
Abundance **(A)** and total abundance **(B)** of genes involved in inorganic P-solubilization and organic P-mineralization in soil with and without P input determined by PICRUSt2. The gene abundance was estimated base on 80,000 reads per sample. Asterisks indicate significant differences between −P and +P treatments (Kruskal–Wallis rank sum test, ^*^p < 0.05, ^**^p < 0.01).

Ten genes involved in P mobilization, including Pi solubilization and Po mineralization, were successfully predicted using PICRUS2 (Figure 2A). Five genes—*ppa* (encoding inorganic pyrophosphatase), *phoD* (encoding alkaline phosphatase D), *phnW* (encoding 2-aminoethylphosphonate-pyruvate transaminase), *phnX* (encoding phosphonoacetaldehyde hydrolase), and *phnA* (encoding phosphonoacetate hydrolase)—were significantly enriched in −P treatment compared with +P treatment, while two genes—*gcd* (encoding quinoprotein glucose dehydrogenase) and *appA* (encoding 4-phytase/acid phosphatase)—were diluted (Figure 2A).

Although the individual genes responded differently to P addition, the total abundance of P-mobilization genes was 12.18% higher in −P treatment compared with that in +P treatment (Figure 2B).

### Pi-solubilizing ability and potential Pi-solubilizing taxa in rhizosphere soil

**Figure 3:**
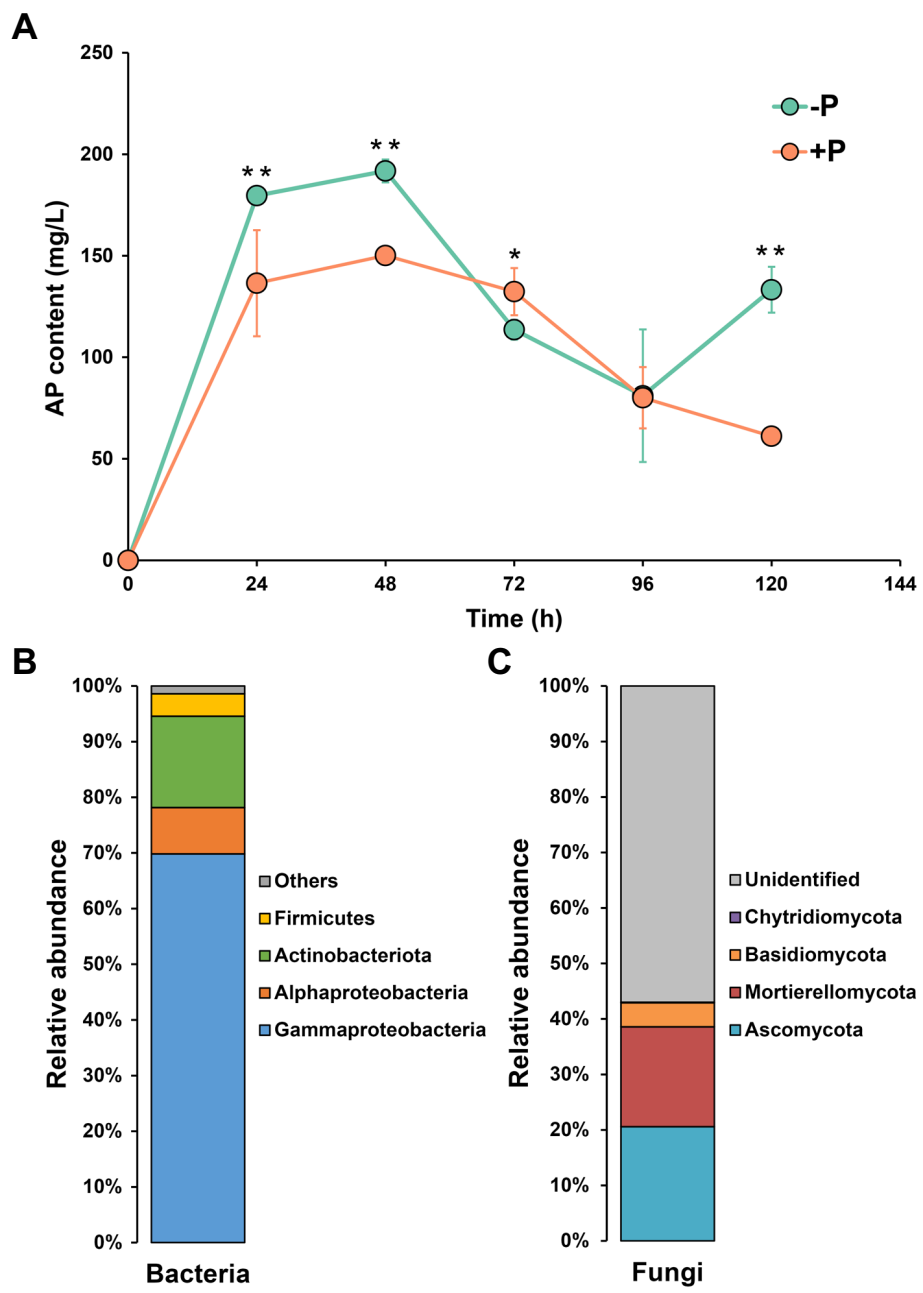
Dynamics of inorganic P-solubilization by microbes in −P and +P treatments **(A)**, and community composition of potential inorganic P-solubilizing bacteria **(B)** and fungi **(C)**. Asterisks indicate significant differences between −P and +P treatments (Kruskal–Wallis rank sum test, ^*^p < 0.05, ^**^p < 0.01).

During the 144-h incubation, the content of dissolved P showed an approximate bell curve (Figure 3), peaking at 48 h. The microbial community from the −P treatment showed a higher capacity of P solubilization compared with the microbial community from the +P treatment. At 48 h, the content of dissolved P in the microbial culture from −P treatment was 1.28-fold that in the culture from +P treatment.

Proteobacteria (including Gammaproteobacteria and Alphaproteobacteria) was the dominant bacterial phylum, accounting for 78.15% of the bacterial community, followed by Actinobacteriota (16.41%), and Firmicutes (4.04%). In the fungal community, 57.01% of the reads could not be identified. Ascomycota, Mortierellomycota, Basidiomycota, and Chytridiomycota were the four identified phyla, which accounted for 20.59, 17.97, 4.34, and 0.09% of the fungal community.

### Variation in Pi-solubilizing agents in the rice rhizosphere microbial community under different P treatments

**Figure 4:**
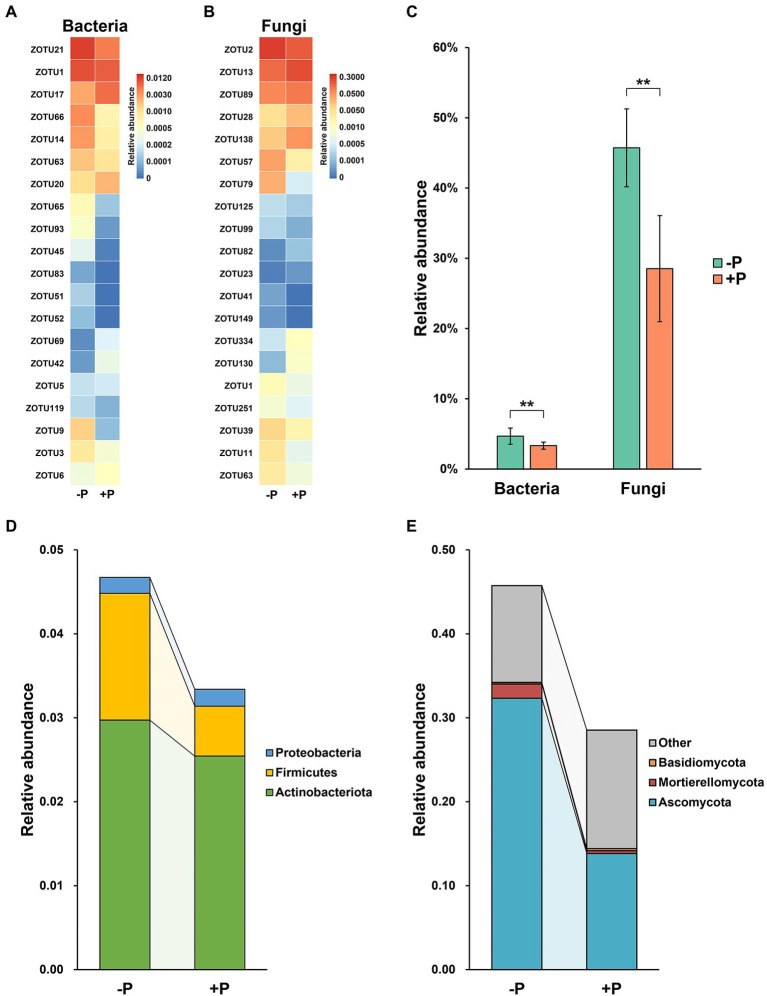
Variation in relative abundance of potential inorganic P-solubilizing ZOTUs in bacterial and fungal communities under −P and +P treatment. Variation among the top 20 ZOTUs in relative abundance of bacterial **(A)** and fungal **(B)** communities. Total relative abundance **(C)** and taxonomic composition of potential Pi-solubilizing OTUs in bacterial **(D)** and fungal **(E)** communities under −P and +P treatment. Asterisks indicate significant differences between −P and +P treatments (Kruskal–Wallis rank sum test, ^**^p < 0.01).

Selection of ZOTUs with potential P-solubilization activity in rhizosphere microbial communities revealed marked variation in these Pi-solubilizing agents under the two different P treatments (Figure 4). Most of the P-solubilizing ZOTUs (including bacteria and fungi) were enriched under −P treatment (Figures 4A,B).

In total, Pi-solubilizing ZOTUs accounted for 4.67% of the bacterial community under −P treatment, which was significantly higher than the proportion in +P treatment (3.33%; Figure 4C). For the fungal community, the relative abundance of Pi-solubilizing ZOTUs was 45.73 and 28.53% in −P and +P treatments, respectively (Figure 4C), and the difference between the two was statistically significant.

Bacterial Pi-solubilizing agents in the rice rhizosphere were assigned as Actinobacteriota, Firmicutes, and Proteobacteria. Firmicutes was the major contributor responsible for the increase in Pi-solubilizing agents in the bacterial community, with a relative abundance that was significantly higher in −P treatment compared with that in +P treatment. In contrast, the relative abundances of Actinobacteriota and Proteobacteria exhibited limited variation between −P and +P treatments (Figure 4D). Most of the fungal Pi-solubilizing agents were identified as Ascomycota, Mortierellomycota, and Basidiomycota, in addition to some unidentified ones. Ascomycota was the dominant fungal Pi-solubilizing agent in −P treatment but was largely diluted in +P treatment (Figure 4E).

### Microbial community assembly under different P treatments

**Figure 5:**
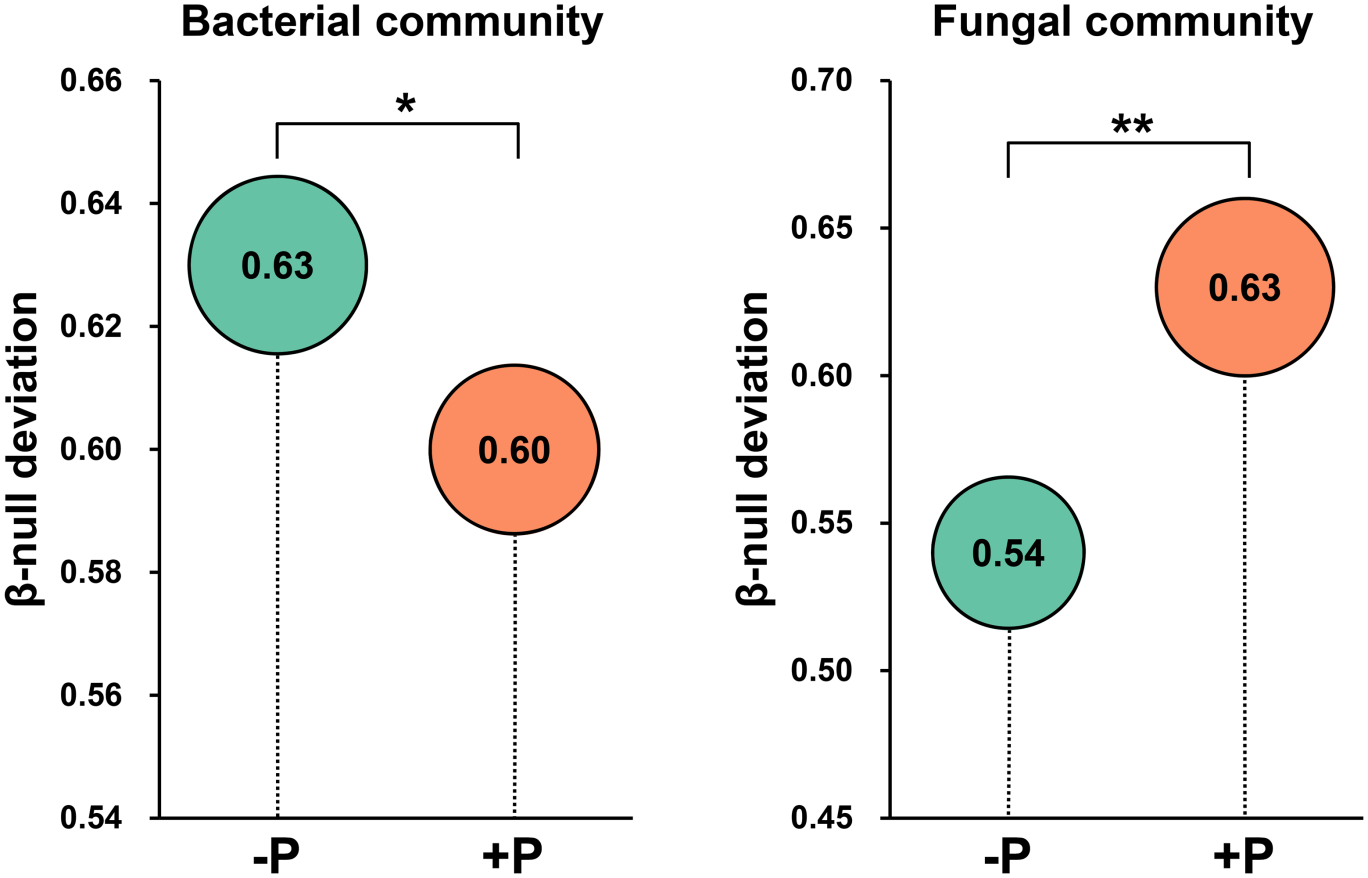
Abundance-based β-null deviation for bacterial and fungal communities based on Bray–Curtis distance. Asterisks indicate a significant difference between treatments as determined by Kruskal–Wallis rank sum test (^*^p < 0.05; ^**^p < 0.01).

Beta-null deviation values for both bacterial and fungal communities were positive, suggesting that the microbial community was dissimilar between patches than expected under the null model of random assembly (Figure 5). However, the beta-null deviation values were significantly different between −P and +P treatments, and the patterns were different for bacterial and fungal communities. The beta-null deviation value was significantly higher in −P treatment compared with that in +P treatment for the bacterial community, while it was significantly lower in −P treatment compared with that in +P treatment for the fungal community (Figure 5). These results demonstrated that the contribution of deterministic assembly processes in the bacterial community was strengthened by P deficiency, while that in the fungal community was weakened by P deficiency.

## Discussion

### Enrichment of P-mobilizing microbes in rice rhizosphere under P deficiency

Rhizosphere microbes play a vital role for plants to mitigate environmental pressures and increase nutrient uptake. In the present study, under P deficiency, bacterial and fungal species involved in P mobilization, including Pi dissolution and Po mineralization, were significantly enriched in rice rhizosphere. This suggests that regulating the rhizosphere microbial community is a potential mechanism through which rice addresses P deficiency. Functional prediction revealed the types of functional genes involved in Pi dissolution and Po mineralization. However, these genes responded differently to P deficiency. For example, *ppa*, *ppx*, and *gcd* are all involved in Pi dissolution, but *ppa* was enriched under P deficiency, while *gcd* was diluted, and *ppx* showed no significant response to P deficiency (Figure 2A). This result differed from those of other studies focused on non-rhizosphere soil. Dai et al. (2020) found that long-term P input did not significantly impact the total relative abundance of genes involved in P mobilization. These differences show the selective effects of root on the functional microbiome in rice rhizosphere.

Pyrophosphate is ubiquitous in soil and is closely associated with many metabolic processes in both plants and microorganisms (Pérez-Castiñeira et al., 2021). However, the role of pyrophosphate in plant nutrition is not fully understood (Reitzel and Turner, 2014). The increase in abundance of the *ppa* gene (encoding inorganic pyrophosphatase) in the rhizosphere under P deficiency indicates that selective recruitment of pyrophosphate hydrolysis bacteria may be a potential way for rice to mitigate P deficiency.

Po is also a major P fraction in soil (Weihrauch and Opp, 2018), accounting for approximately 50% of the P pool in some studied soils (Yan et al., 2020), and Po mineralization is another way to increase P bioavailability. However, in the current study, the genes involved in Po mineralization also responded differently to P deficiency (Figure 2A), indicating the different effects of rice roots on these functional microbes. The selective effects of rice roots on functional rhizosphere microbial communities under P deficiency was also reflected by the changes in the taxonomic composition of the Pi-solubilizing bacterial community (Figures 4A–E). Types of microorganisms were identified in the studied soil (Figures 3B,C), and the total relative abundance of these microorganisms were significantly higher in −P treatment compared with that in +P treatment. However, the change sizes of these microorganisms were not equal, or even homodromous, suggesting that although these microorganisms have the same function in P mobilization, their associations with rice are largely different. This may be attributed to the different preference of microorganisms for substrate type. There are various P fractions in soil, however, microorganisms have different capacity and mechanisms in dissolution or mineralization of these fractions (Rofner et al., 2016; Alori et al., 2017; Chen and Liu, 2019). Furthermore, plants may prefer to recruit microorganisms with high P-mobilizing capacity to obtain high efficiency in mitigating P deficiency. In addition, some P-mobilizing strains are also plant growth-promoting rhizobacteria (PGPR), which promote plant growth in many ways, such as through producing phytohormones and controlling phytopathogens. Phosphate solubilization by rhizosphere microorganisms was found to be coupled with production of phytohormones, such as indole-3-acetic acid (IAA) and gibberellic acid (GA; Adhya et al., 2015; Kalayu, 2019; Song et al., 2022). Thus, plants may prefer to recruit these PGPR rather than those that only have P-mobilization capacity.

Both bacteria and fungi are vital for plant P uptake; however, their contribution to P mobilization in the acidic soil of the current study appeared markedly different. In rice rhizosphere, the Pi-solubilizing bacteria accounted for only 3.33–4.67% of the total bacterial community, while this proportion was 28.52–45.73% in the fungal community (Figures 4D,E). This may indicate that fungi are the dominant contributor for Pi dissolution in the studied soil. In addition, our previous study showed that fungi had a higher capability for Po mineralization compared with bacteria in the studied soil (Chen et al., 2020). Taken together, these results suggested that in the acidic soil, P availability may be greatly regulated by fungi, and this may be partly associated with the differing resistance of bacteria and fungi to soil acidity. In general, the soil bacterial community was more sensitive to soil pH compared with the fungal community. Close correlation between soil bacterial communities and pH has been widely observed, including in natural systems and agricultural systems (Lauber et al., 2009; Rousk et al., 2010; Shen et al., 2013; Sun et al., 2015). However, fungi generally exhibit wider pH ranges for optimal growth compared with bacteria (Rousk et al., 2010). Thus, fungi may be more active in P mobilization in acidic soil compared with bacteria. However, as a culturable approach was used in this study, there are some bias for the comparison of the contribution of bacteria and fungi to P solubilization, as well as for the identification of the microbial taxa involved in P solubilization. More works are still needed to accurately quantify the contribution of bacteria and fungi in P solubilization and identify the functional microbial microflora.

### Variations in community assembly of bacteria and fungi in rice rhizosphere under P deficiency

Understanding the mechanisms of microbial community assembly in the rhizosphere is a crucial topic in the study of interactions between microorganisms and plants. In this study, the assembly of bacterial and fungal communities in rice rhizosphere responded differently to P deficiency. Beta-null deviation analysis showed that the contribution of deterministic processes in governing the bacterial community was significantly increased under P deficiency, but was largely decreased in shaping the fungal community (Figure 5). This indicated that the recruitment of rhizosphere microorganisms by rice was greatly influenced by P concentration in the soil. This differing impact of P input on rice root-associated bacterial and fungal communities has been observed in other studies (Long and Yao, 2020). The current study further revealed that under P deficiency, roots seemed to strengthen the filter effect on the bacterial community but weakened the filter effect on the fungal community. Hence, lower bacterial diversity and higher fungal diversity were observed under P deficiency (Figures 1A,B). Although both bacterial and fungal Pi-solubilizing agents were enriched in rice rhizosphere under P deficiency, rice may prefer to recruit fungi to mitigate P deficiency.

Secretion of exudates was considered the primary approach for plants to regulate rhizosphere microbiomes (Xing-Feng et al., 2014). The type and amount of rice root exudate varies greatly under P deficiency. Hoffland et al. (2006) found that rice exudation rates of citrate and oxalate significantly increased under P deficiency. Tawaraya et al. (2018) detected 99 metabolites in rice root exudates, and the concentration of betaine, gamma-aminobutyric acid, and glutarate were higher at a low P level, while that of spermidine was lower at a low P level. Some of these exudates could directly mobilize soil P to increase its bioavailability, while also modifying the rhizosphere microbial community. In this study, lower pH was observed in rhizosphere soil of rice under −P treatment (Table 1), indicating higher secretion of acid exudates under P deficiency. In addition, exudates from rice root under −P treatment had higher Pi-solubilizing ability than that under +P treatments (Supplementary Figure S1). In addition, root exudates significantly enhancing microbial Pi-solubilizing ability, and the effects of exudates from rice root under −P treatment was significantly higher than that under +P treatment (Supplementary Figure S1). These results confirmed the great effects of root exudates in increasing nutrient bioavailability through direct mobilization and indirect modification of microbial functions. However, the establishment of the rhizosphere microbial community might be determined by both the chemical composition of root exudates and by microbial substrate preferences (Ding et al., 2019). Thus, the root exudates usually selectively recruit the rhizosphere microorganisms and shape a unique rhizosphere microbial community under different environments. For example, Kim et al. (2022) reported that rice root exudate orobanchol enriched Acidobacteria that potentially solubilize phosphate, while 4-deoxyorobanchol was associated with the genera *Dyella* and *Umbelopsis*. The different assembly of bacterial and fungal communities in rice rhizosphere under P deficiency indicated the selective effects of rice root exudates in recruiting bacterial and fungal communities. Further work is needed to determine which exudates are responsible for the effects and the mechanisms of action of these exudates.

## Conclusion

Under P deficiency, bacterial and fungal communities in rice rhizosphere were great changed compared with that without P deficiency, and both P-mobilizing bacteria and fungi were enriched, but the proportion of P-mobilizing agents in the bacterial community was significantly lower than that of the fungal community. In addition, the contribution of deterministic processes to bacterial community assembly was increased under P deficiency, but was decreased in relation to fungal community assembly. These results revealed the stronger selectively filtering effects from rice root on the bacterial community and weaker selectively filtering effects on the fungal community, which accounted for the decrease in bacterial diversity and increase in fungal diversity, and also suggested that rice tends to recruit a wider variety of fungi to respond to P deficiency. All the findings from the study indicated the different recruiting strategies for bacterial and fungal communities and the marked contribution of fungi in P mobilization in rice rhizosphere under P deficiency.

## Data availability statement

The datasets presented in this study can be found in online repositories. The names of the repository/repositories and accession number(s) can be found at: https://www.ebi.ac.uk/ena, PRJEB52853.

## Author contributions

RS, XX, and XS designed the experiment. BL provided experimental materials and directed the experiment. WZ and WY performed laboratorial measurement. RS, WZ, RC, CZ, and XX performed data analysis. RS, XS, YL, and CZ wrote the paper. All authors contributed to the article and approved the submitted version.

## Funding

This work was supported by the Key Science and Technology Project of Anhui Province (202103a06020012), the Nature Science Foundation of Anhui Province (2108085QC123), and the Hebei Technology Innovation Center for Green Management of Soil-borne Diseases (Baoding University) (2021K08).

## Conflict of interest

The authors declare that the research was conducted in the absence of any commercial or financial relationships that could be construed as a potential conflict of interest.

## Publisher’s note

All claims expressed in this article are solely those of the authors and do not necessarily represent those of their affiliated organizations, or those of the publisher, the editors and the reviewers. Any product that may be evaluated in this article, or claim that may be made by its manufacturer, is not guaranteed or endorsed by the publisher.
