# Supplementary material for: Changes in phosphorus mobilization and community assembly of bacterial and fungal communities in rice rhizosphere under phosphate deficiency
Source: Front Microbiol. 2022 Aug 3;13:953340. doi: 10.3389/fmicb.2022.953340 (PMC9382406; doi:10.3389/fmicb.2022.953340)
Supplement: Supplementary file 1 [file Data_Sheet_1.DOCX]

**Supporting Information**

**Changes in phosphorus mobilization and community assembly of bacterial and fungal communities in rice rhizosphere under phosphate deficiency**

Ruibo Sun^1, 2, †^, Wenjie Zhang^1, 2, †^, Yangbing Liu^3^, Yun Wenjing^1, 2^, Bingbing Luo^1, 2^, Rushan Chai^1, 2^, Chao Ma^1, 2^, Chaochun Zhang^1, 2^, Xingjia Xiang^4, *^, Xiaofeng Su^5,^ *

^1^ Anhui Province Key Lab of Farmland Ecological Conservation and Pollution Prevention; Engineering and Technology Research Center of Intelligent Manufacture and Efficicent Utilization of Green Phosphorus Fertilizer of Anhui Province, College of Resources and Environment, Anhui Agricultural University, No.130 Changjiangxilu, Hefei, 230036, P. R. China.

^2^ Key Laboratory of JiangHuai Arable Land Resources Protection and Eco-restoration, Ministry of Natural Resources, College of Resources and Environment, Anhui Agricultural University, No.130 Changjiangxilu, Hefei, 230036, P. R. China.

^3^ Anhui Provincial Territorial Space Planning Institute, No.302 Fanhua Avenue, Hefei 230601, China

^4^ Anhui Province Key Laboratory of Wetland Ecosystem Protection and Restoration, School of Resources and Environmental Engineering, Anhui University, No.11 Jiulong Road, Hefei, 233601, China

^5^ Biotechnology Research Institute, Chinese Academy of Agricultural Sciences, No.12 Zhongguancun South Street, Beijing 100081, China

**Collection of root exudates**

Root exudates were collected using SOIL-HYDROPONIC-HYBRID approach (Oburger and Jones, 2018). Plant was taken out from soil carefully, then roots were washed with sterile water to remove soil. The plant was placed into a baffled flask with 100 ml sterile double distilled water, and incubated for 30 min. Then the culture water was filtered by using 0.45mm filter paper. The percolate was used for downstream experiment.

**Impact of root exudates on microbial Pi-solubilizing capacity**

Soil microbes (SM) were extracted from 1 g soil with 100 ml sterile water, then 5 mL supernatant was added to 50 mL sterile PVK liquid medium for incubation. Five treatments were set up, including Control (PVK with SM), RE- (PVK with 5 ml root exudates from -P treatments), RE+ (PVK with 5 ml root exudates from +P treatments), MRE- (RE- with SM), and MRE+ (RE+ with SM). All the culture systems were adjusted to 60 ml with sterile water. Each treatment contained three replicates. All the culture systems were incubated for 2 days at 28°C and 180 rpm in a thermostatic shaker. After incubation, the culture was centrifuged at 5000 *g* for 2 min. P content in the supernatant was measured.

**Reference**

Oburger, E., and Jones, D.L. (2018). Sampling root exudates – Mission impossible? Rhizosphere 6, 116-133. doi: 10.1016/j.rhisph.2018.06.004.


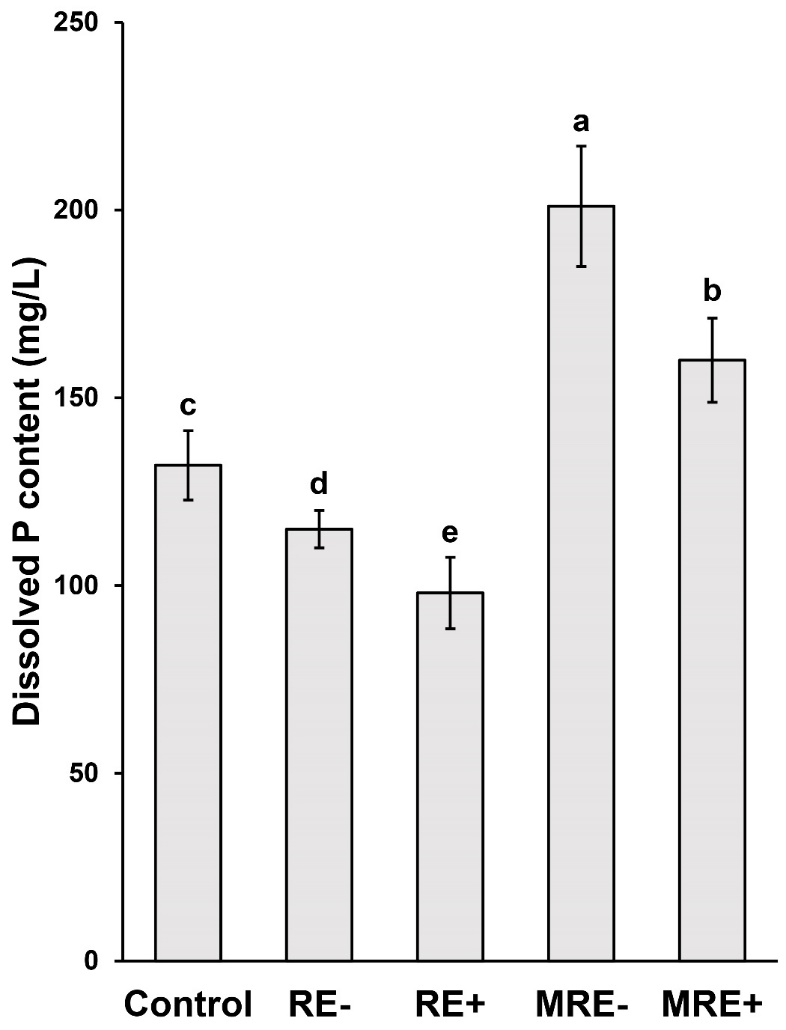


**Figure S1. Dissolved P in the culture systems**

Control: PVK with SM; RE-: PVK with root exudates from -P treatments; RE+: PVK with root exudates from +P treatments; MRE-: RE- with SM, MRE+: RE+ with SM.

Different letters indicate signiﬁcant difference between treatments as determined by Kruskal–Wallis rank sum test (*p* < 0.05).
